# Bosentan reverses the pro-fibrotic phenotype of systemic sclerosis dermal fibroblasts via increasing DNA binding ability of transcription factor Fli1

**DOI:** 10.1186/ar4529

**Published:** 2014-04-03

**Authors:** Kaname Akamata, Yoshihide Asano, Naohiko Aozasa, Shinji Noda, Takashi Taniguchi, Takehiro Takahashi, Yohei Ichimura, Tetsuo Toyama, Shinichi Sato

**Affiliations:** 1 Department of Dermatology, University of Tokyo Graduate School of Medicine, 7-3-1 Hongo, Bunkyo-ku, Tokyo 113-8655, Japan

## Abstract

**Introduction:**

Although the pathogenesis of systemic sclerosis (SSc) still remains unknown, recent studies have demonstrated that endothelins are deeply involved in the developmental process of fibrosis and vasculopathy associated with SSc, and a dual endothelin receptor antagonist, bosentan, has a potential to serve as a disease modifying drug for this disorder. Importantly, endothelin-1 (ET-1) exerts a pro-fibrotic effect on normal dermal fibroblasts and bosentan reverses the pro-fibrotic phenotype of SSc dermal fibroblasts. The purpose of this study was to clarify the details of molecular mechanisms underlying the effects of ET-1 and bosentan on dermal fibroblasts, which have not been well studied.

**Methods:**

The mRNA levels of target genes and the expression and phosphorylation levels of target proteins were determined by reverse transcription real-time PCR and immunoblotting, respectively. Promoter assays were performed using a sequential deletion of human α2 (I) collagen (COL1A2) promoter. DNA affinity precipitation and chromatin immunoprecipitation were employed to evaluate the DNA binding ability of Fli1. Fli1 protein levels in murine skin were evaluated by immunostaining.

**Results:**

In normal fibroblasts, ET-1 activated c-Abl and protein kinase C (PKC)-δ and induced Fli1 phosphorylation at threonine 312, leading to the decreased DNA binding of Fli1, a potent repressor of the *COL1A2* gene, and the increase in type I collagen expression. On the other hand, bosentan reduced the expression of c-Abl and PKC-δ, the nuclear localization of PKC-δ, and Fli1 phosphorylation, resulting in the increased DNA binding of Fli1 and the suppression of type I collagen expression in SSc fibroblasts. In bleomycin-treated mice, bosentan prevented dermal fibrosis and increased Fli1 expression in lesional dermal fibroblasts.

**Conclusions:**

ET-1 exerts a potent pro-fibrotic effect on normal fibroblasts by activating “c-Abl - PKC-δ - Fli1” pathway. Bosentan reverses the pro-fibrotic phenotype of SSc fibroblasts and prevents the development of dermal fibrosis in bleomycin-treated mice by blocking this signaling pathway. Although the efficacy of bosentan for dermal and pulmonary fibrosis is limited in SSc, the present observation definitely provides us with a useful clue to further explore the potential of the upcoming new dual endothelin receptor antagonists as disease modifying drugs for SSc.

## Introduction

Systemic sclerosis (SSc) is a multisystem connective tissue disease characterized by immune abnormalities, vascular injuries, and fibrosis of skin and certain internal organs [1]. Although the pathogenesis of SSc still remains unclear, it has been believed that fibroblast activation is a final consequence following inflammation, autoimmune attacks, and vascular damage. A wealth of evidence suggests that, once activated, SSc dermal fibroblasts establish a self-activation system by autocrine transforming growth factor (TGF)-β stimulation at least partially via upregulating cell surface receptors for latent-form TGF-β, such as integrin αVβ3, integrin αVβ5, and thrombospondin-1 [2-6]. A plausible strategy to treat fibrosis in SSc is to block the autocrine TGF-β signaling in SSc fibroblasts and better understanding of its molecular mechanism is necessary to develop the treatment for this complicated disorder [1,7].

The endothelins are a family of 21-amino-acid peptides mainly produced by endothelial cells, which consist of three isoforms, including endothelin-1 (ET-1) and the related peptides endothelin-2 and -3. In addition to a potent vasoconstrictive effect, ET-1 possesses a wide range of biological effects on different cell types. Several lines of evidence have demonstrated that ET-1 plays a pivotal role in the process of fibroblast activation as a downstream target of TGF-β [8-11]. TGF-β1 induces the expression of ET-1 through Smad- and activator protein-1/c-Jun N-terminal kinase-dependent signaling in human dermal fibroblasts, while through a Smad-independent ALK5/activator protein-1/c-Jun N-terminal kinase-dependent signaling in human lung fibroblasts, and the ability of TGF-β1 to trigger the pro-fibrotic gene program is dependent on ET-1 in both of these cells [9,12]. In animal models, forced expression of ET-1 accelerates dermal wound healing as well as TGF-β1, while blockade of endothelin signaling with bosentan, a dual endothelin receptor antagonist, significantly inhibits the effect of TGF-β1 on dermal wound healing [12]. Importantly, bosentan also attenuates bleomycin (BLM)-induced skin fibrosis in animal models [12]. Thus, endothelins are potentially implicated in the pathogenesis of fibrotic disorders.

The role of ET-1 has also been well-studied in SSc. Circulating ET-1 levels are elevated in diffuse cutaneous SSc and limited cutaneous SSc patients compared with healthy controls, and in limited cutaneous SSc patients with pulmonary arterial hypertension as compared to those without [13,14], suggesting the involvement of ET-1 in the development of fibrotic and vascular involvement associated with SSc. Clinically, bosentan has been shown to prevent the development of new digital ulcers in SSc patients by two high-quality randomized clinical trials [15,16]. As for pulmonary arterial hypertension associated with SSc, bosentan mostly prevents the exacerbation of hemodynamic parameters and improves exercise tolerance evaluated by 6-minute walk distance [17-19]. These clinical data suggest that bosentan is useful for the treatment of vascular involvement in SSc. In contrast, the clinical efficacy of bosentan for interstitial lung disease (ILD) is relatively limited [20,21], though bosentan reverses the pro-fibrotic phenotype of cultured SSc lung fibroblasts [8-11]. Since this discrepancy between experimental data and clinical observation may be partly due to the relatively lower tissue distribution of bosentan (approximately 1%) [22], endothelin receptor antagonists with better tissue distribution have a potential to improve the pathological fibrosis in SSc.

As described above, although ET-1 is a potent pro-fibrotic peptide and bosentan exerts a novel anti-fibrotic effect, the detailed molecular mechanism explaining these observations has still remained unknown. Therefore, we herein investigated the mechanisms by which ET-1 drives a pro-fibrotic gene program in normal dermal fibroblasts and bosentan reverses the pro-fibrotic phenotype of SSc dermal fibroblasts and the experimental dermal fibrosis in animal models.

## Methods

### Reagents

Anti-Fli1 antibody for immunoblotting and immunofluorescence and anti-protein kinase C (PKC)-δ antibody were purchased from Santa Cruz Biotechnology (Santa Cruz, CA, USA). Anti-Fli1 antibody for immunohistochemistry was obtained from BD Biosciences (San Diego, CA, USA). Antibodies against c-Abl and phospho-c-Abl (Tyr245) were bought from Cell Signaling (Danvers, MA, USA). Antibodies for β-actin and α-smooth muscle actin (α-SMA) and antibody for type I collagen were products from Sigma-Aldrich (St. Louis, MO) and Southern Biotech (Birmingham, AL, USA), respectively. The polyclonal rabbit anti-phospho-Fli1 (Thr312)-specific antibody was generated as described previously [23]. Bosentan was a gift from Actelion Pharmaceuticals (Allschwil, Switzerland). Smad3 inhibitor SIS3 was purchased from Calbiochem (San Diego, CA, USA).

### Cell cultures

Human dermal fibroblasts were obtained by skin biopsy from the affected areas (dorsal forearm) of eight patients with diffuse cutaneous SSc with less than 2 years of skin thickening and from the corresponding area of eight closely matched healthy donors. Fibroblasts were cultured in Dulbecco’s modified eagle medium with 10% fetal calf serum, 2 mM L-glutamine, and the antibiotic antimycotic solution. These cells were individually maintained as monolayers at 37°C in 95% air, 5% CO_2_. All studies used cells from passage number three to six. The study was performed according to the Declaration of Helsinki and approved by the ethical committee of the University of Tokyo Graduate School of Medicine. Written informed consent was obtained from all of the patients and healthy controls.

### Cell viability assay

Cell viability was evaluated by the trypan blue exclusion test. Cells were treated with the indicated concentration of bosentan. Cell viability was examined at 24 and 48 hours. Stained (dead) and unstained (viable) cells were counted with a hematocytometer.

### Immunoblotting

Confluent quiescent fibroblasts were serum-starved for 48 hours and harvested. In some experiments, cells were stimulated with ET-1 or bosentan for the indicated period of time before being harvested. Whole cell lysates and nuclear extracts were prepared as described previously [2,24]. Samples were subjected to sodium dodecyl sulfate-polyacrylamide gels electrophoresis and immunoblotting with the indicated primary antibodies. Bands were detected using enhanced chemiluminescent techniques (Thermo Scientific, Rockford, IL, USA). According to a series of pilot experiments, anti-Fli1 antibody and anti-phospho-Fli1 (Thr312)-specific antibody work much better in immunoblotting using nuclear extracts and whole cell lysates, respectively.

### RNA isolation and reverse transcription (RT) real-time PCR

Total RNA was isolated from normal and SSc fibroblasts with RNeasy spin columns (Qiagen, Crawley, UK). One μg of total RNA from each sample was reverse-transcribed into cDNA using the iScript cDNA synthesis kit (Bio-Rad, Hercules, CA, USA). Real-time quantitative PCR was performed using Fast SYBR Green PCR Master Mix (Applied Biosystems, Carlsbad, CA, USA) on ABI prism 7000 (Applied Biosystems) in triplicates. Human α2 (I) collagen (COL1A2) and Fli1 mRNA levels were normalized to human 18S rRNA mRNA levels. The sequences of primers for COL1A2, Fli1, and 18S rRNA were obtained from previous publications [25,26]. The ΔΔCt method was used to compare target gene and housekeeping gene (18S rRNA) mRNA expression.

### Plasmid construction

Generation of a series of 5′-deletions of COL1A2/chloramphenicol acetyltransferase (CAT) construct consisting of the *COL1A2* gene fragments (+58 to −353, −264, −186, or −108 bp relative to the transcription start site) linked to the CAT reporter gene was done as previously described [27,28].

### The evaluation of COL1A2 promoter activity by RT real-time PCR

Normal or SSc fibroblasts were grown to 50% confluence in 100-mm dishes, transfected with the indicated constructs along with pSV-β-galactosidase (β-GAL) using FuGENE6 (Roche Diagnostics, Indianapolis, IN, USA). After overnight incubation at 37°C, some cells were further stimulated with ET-1 or bosentan for 24 hours. The cells were harvested and CAT and β-GAL mRNA levels were determined using RT real-time PCR. Transfection efficiency was normalized by β-GAL mRNA levels. In some samples, it was confirmed that this method reproduces the results of relative promoter activity evaluated by the canonical method of CAT reporter assay using [^14^C]-chloramphenicol. The sequences of primers were as follows: CAT forward 5′-TTCGTCTCAGCCAATCCCTGGGTGA-3′ and reverse 5′-CCCATCGTGAAAACGGGGGCGAA-3′; β-GAL forward 5′-TCCACCTTCCCTGCGTTA-3′ and reverse 5′-AGAAGTCGGGAGGTTGCTG-3′.

### DNA affinity precipitation assay

DNA affinity precipitation assay was performed as described previously [29]. Briefly, nuclear extracts prepared from dermal fibroblasts were incubated for 10 minutes at 4°C with gel shift binding buffer, and 20 μg of poly (dI-dC) in a final volume of 1 ml. Pre-clearing was performed by adding streptavidin-coupled agarose beads and incubating the mixture for 30 minutes at 4°C. After centrifugation, the supernatant was incubated with 500 pM of COL1A2-EBS oligonucleotide, which corresponds to bp −307 to −269 of COL1A2 promoter, or COL1A2-EBS-M oligonucleotide, which has a mutated Ets binding site (EBS) of COL1A2-EBS oligonucleotide, overnight at 4°C. Then, streptavidin-coated agarose beads were added, followed by a further 2-hour incubation at 4°C. The protein-DNA-streptavidin-agarose complex was washed twice with Tris-ethylenediaminetetraacetic acid (EDTA) including 100 mM NaCl, twice with gel shift binding buffer, and once with PBS. Precipitates were subjected to immunoblotting with anti-Fli1 antibody.

### Chromatin immunoprecipitation (ChIP) assay

ChIP assay was carried out using EpiQuik ChIP kit (Epigentek, Farmingdale, NY, USA). Briefly, cells were treated with 1% formaldehyde for 10 minutes. The cross-linked chromatin was then prepared and sonicated to an average size of 300 to 500 bp. The DNA fragments were immunoprecipitated with anti-Fli1 antibody at 4°C. As a negative control, normal rabbit IgG was used. After reversal of crosslinking, the immunoprecipitated chromatin was quantified by RT real-time PCR. The primers were as follows: COL1A2/F-404, 5′-CTGGACAGCTCCTGCTTTGAT-3′; COL1A2/R-233, 5′-CTTTCAAGGGGAAACTCTGACTC-3′.

### BLM-induced murine model of SSc

BLM (Nippon Kayaku, Tokyo, Japan) or PBS was injected subcutaneously into the back of C57BL/6 mice daily for 3 weeks, as described previously [30].

### Immunohistochemistry

Immunohistochemistry with Vectastain ABC kit (Vector Laboratories, Burlingame, CA, USA) was performed on formalin-fixed, paraffin-embedded tissue sections using anti-Fli1 antibody according to the manufacturer’s instruction.

### Immunofluorescence

Rabbit anti-Fli1 antibody and mouse anti-α-SMA antibody were used as primary antibodies and Alexa Fluor 546 goat anti-rabbit IgG antibody (Invitrogen, Carlsbad, CA, USA) and fluorescein isothiocyanate (FITC)-conjugated rat anti-mouse IgG antibody (Roche) were used as secondary antibodies. Coverslips were mounted by using Vectashield with DAPI (Vector Laboratories), and staining was examined by using Bio Zero BZ-8000 (Keyence, Osaka, Japan) at 495 nm (green), 565 nm (red), and 400 nm (blue).

### Statistical analysis

Statistical analysis was carried out with the Mann-Whitney *U*-test to compare the distributions of two unmatched groups. The paired *t*-test was used for the comparison of paired data after confirming the normal distribution. Statistical significance was defined as a *P*-value of <0.05.

## Results

### ET-1 rapidly induced the expression of type I collagen via the non-Smad signaling pathway

**Figure 1:**
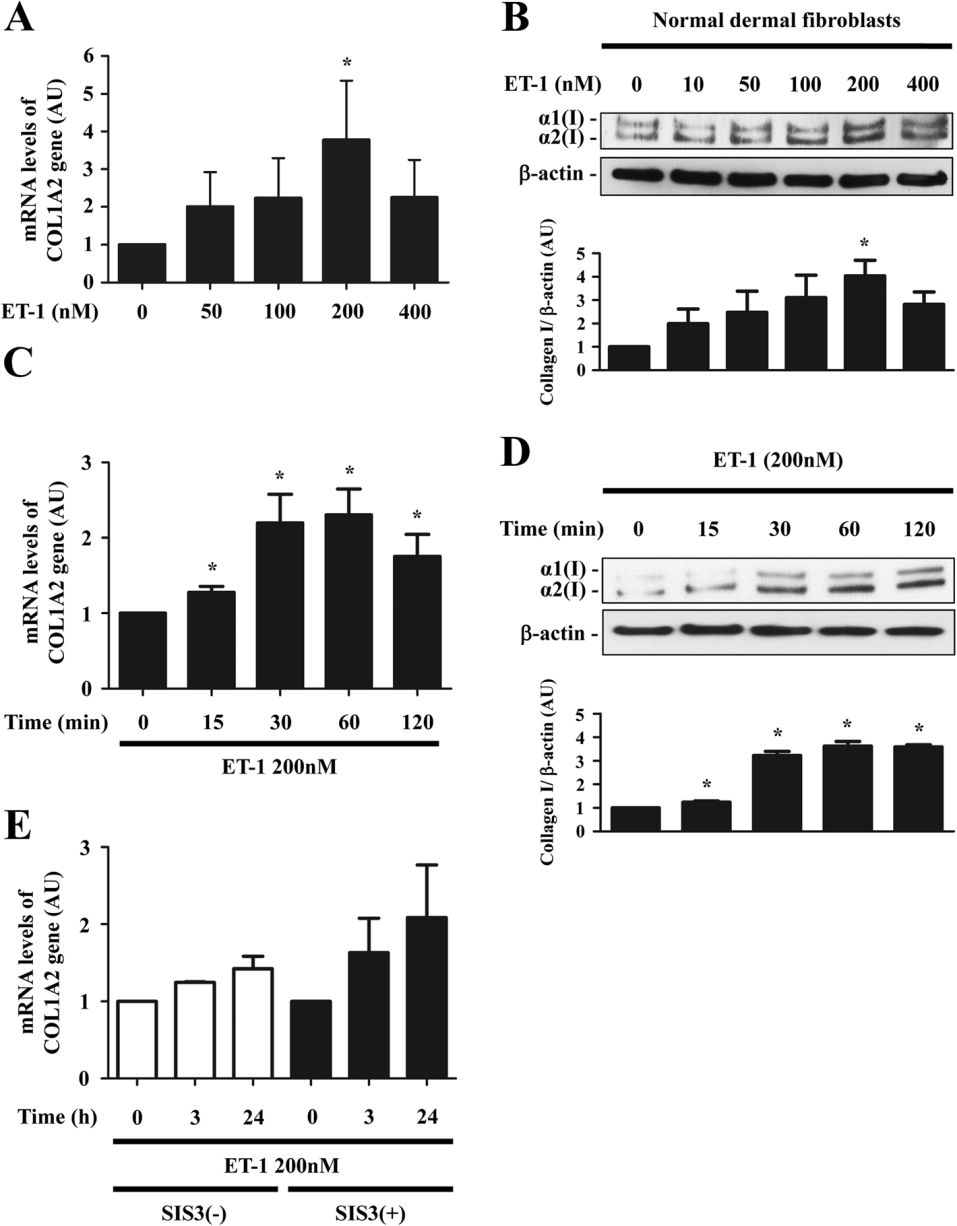
**Endothelin-1 (ET-1) induced the expression of type I collage through the non-Smad pathway in normal dermal fibroblasts. A**, **B**. Quiescent normal dermal fibroblasts were treated with the indicated concentration of ET-1 for 24 hours. mRNA levels of the human α2 (I) collagen (*COL1A2*) gene were determined by reverse transcription (RT) real-time PCR **(A)**. Under the same condition, whole cell lysates were subjected to immunoblotting with anti-type I collagen antibody **(B)**. **(C, D)** Quiescent normal dermal fibroblasts were treated with 200 nM of ET-1 for the indicated period of time. mRNA levels of the *COL1A2* gene **(C)** and the protein levels of type I collagen in whole cell lysates **(D)** were evaluated by RT real-time PCR and immunoblotting, respectively. **(E)** Quiescent normal dermal fibroblasts were treated with SIS3 (3 μM) or methanol. One hour later, some of the cells were treated with 200 nM of ET-1 for 3 or 24 hours. mRNA levels of the *COL1A2* gene were assessed by RT real-time PCR. The graph represents fold change in mRNA levels of the *COL1A2* gene and protein levels of type I collagen, which were quantified by densitometry, in response to ET-1 in comparison to unstimulated controls, which were arbitrarily set at 1. *P <0.05 versus control cells untreated with ET-1. α1(I), α1(I) procollagen; α2(I), α2(I) procollagen; AU, arbitrary unit.

As an initial experiment, to determine the optimal dose of ET-1 to induce the expression of type I collagen in normal dermal fibroblasts, cells were treated with ET-1 at the concentration of 0, 50, 100, 200, or 400 nM for 24 hours and mRNA levels of the *COL1A2* gene were determined by RT real-time PCR. As shown in Figure 1A, ET-1 significantly increased the mRNA levels of the *COL1A2* gene at the concentration of 200 nM. To further confirm this finding at protein levels, the levels of type I collagen protein were determined by immunoblotting under the same conditions. As shown in Figure 1B, ET-1 induced the expression of type I collagen protein in a dose-dependent manner, reaching a peak at the concentration of 200 nM. Therefore, in the following studies, we stimulated cells with ET-1 at the concentration of 200 nM.

We next investigated the time course of ET-1-dependent induction of type I collagen in normal dermal fibroblasts. As shown in Figure 1C, the increase in the mRNA levels of the *COL1A2* gene was observed modestly but significantly as early as 15 minutes and reached a maximum around 30 to 60 minutes after the ET-1 stimulation. This observation was also reproduced at protein levels by immunoblotting (Figure 1D). As Smad3 has a big impact on the TGF-β-dependent regulation of type I collagen expression, we also investigated the effect of Smad3 inhibitor SIS3 on the ET-1-induced mRNA expression of the *COL1A2* gene. As shown in Figure 1E, SIS3 did not affect the mRNA levels of the *COL1A2* gene induced by ET-1 stimulation. Collectively, these results indicate that ET-1 rapidly increases the expression of type I collagen via the non-Smad signaling pathway.

### The responsive element of COL1A2 promoter to ET-1 was located between −353 and −264 bp, which includes the Fli1 binding site

**Figure 2:**
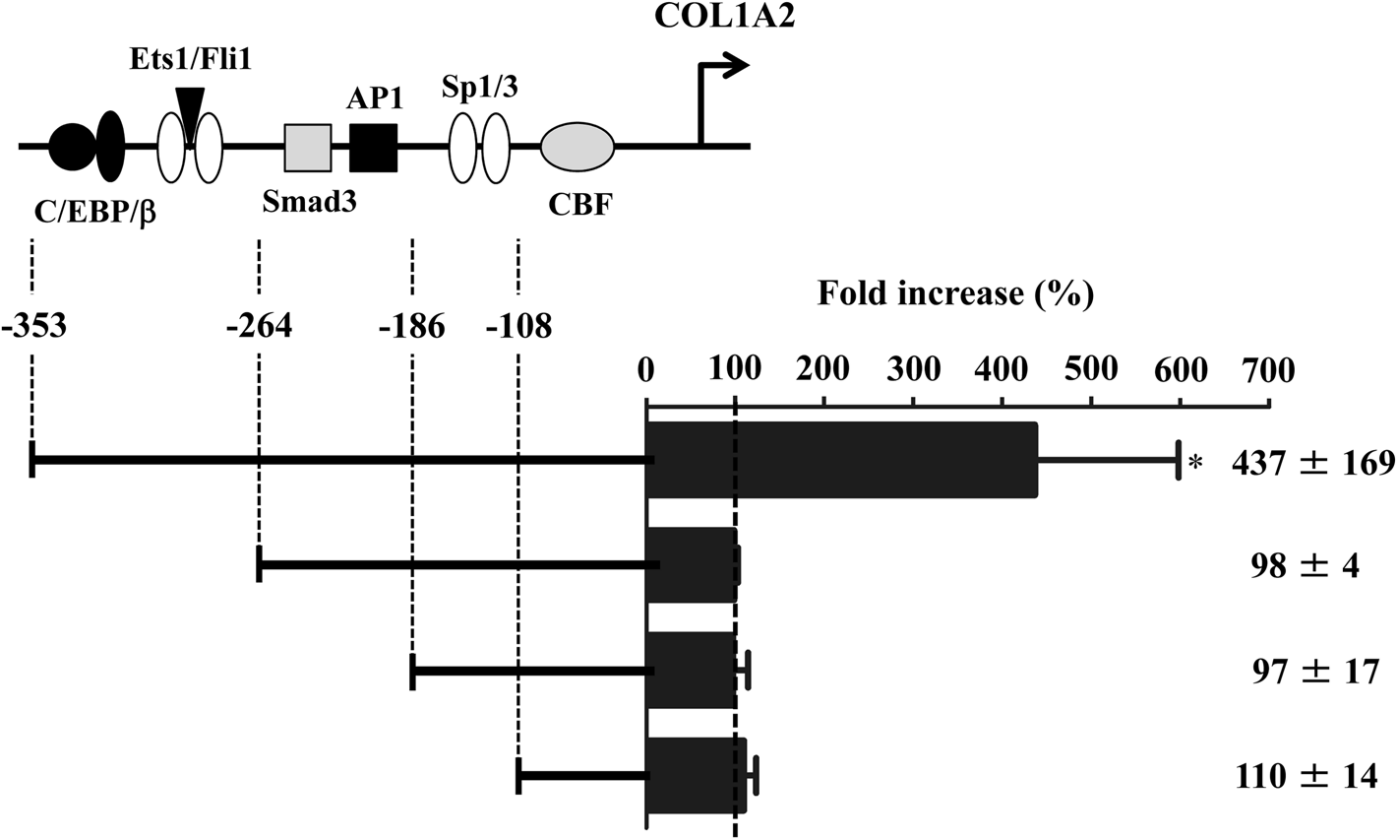
**Identification of the responsive element to endothelin-1 (ET-1) stimulation in the human α2 (I) collagen (COL1A2) promoter.** Normal dermal fibroblasts were transfected with 2 μg of the indicated 5′-deletion of the COL1A2/CAT construct and incubated for 48 hours. In some experiments, cells were treated with ET-1 (200 nM) for the last 24 hours. Values represent CAT mRNA levels relative to those of untreated cells transfected with the same COL1A2/CAT construct (100). The mean and SD from five separate experiments are shown. *P <0.05 versus control cells untreated with ET-1.

To identify potential regulatory elements of the *COL1A2* gene by ET-1, we performed a reporter analysis using a series of 5′-deletions of the COL1A2/CAT construct. As basal promoter activities of these constructs have been well-studied in our previous reports [31], we focused on the difference in the fold increase of each promoter activity induced by ET-1 stimulation. As shown in Figure 2, ET-1 significantly increased the promoter activity of the −353 COL1A2/CAT construct, whereas ET-1 totally lost its stimulatory effect on the promoter activity of the −264, −186, and −108 COL1A2/CAT constructs. These results indicate that the responsive element of COL1A2 promoter to ET-1 is located between −353 and −264 bp. Given that ET-1 increases the mRNA expression of the *COL1A2* gene independently of Smad3, ET-1 potentially exerts its stimulatory effect on the *COL1A2* gene by inactivating transcriptional repressor(s). As the binding site of Fli1, a potent repressor of the *COL1A2* gene, is located at −285 to −282 bp [32], we speculated that Fli1 may play a central role in the ET-1-dependent regulation of the *COL1A2* gene expression.

### ET-1 induced the phosphorylation of Fli1 at threonine 312 and decreased its binding to the COL1A2 promoter

**Figure 3:**
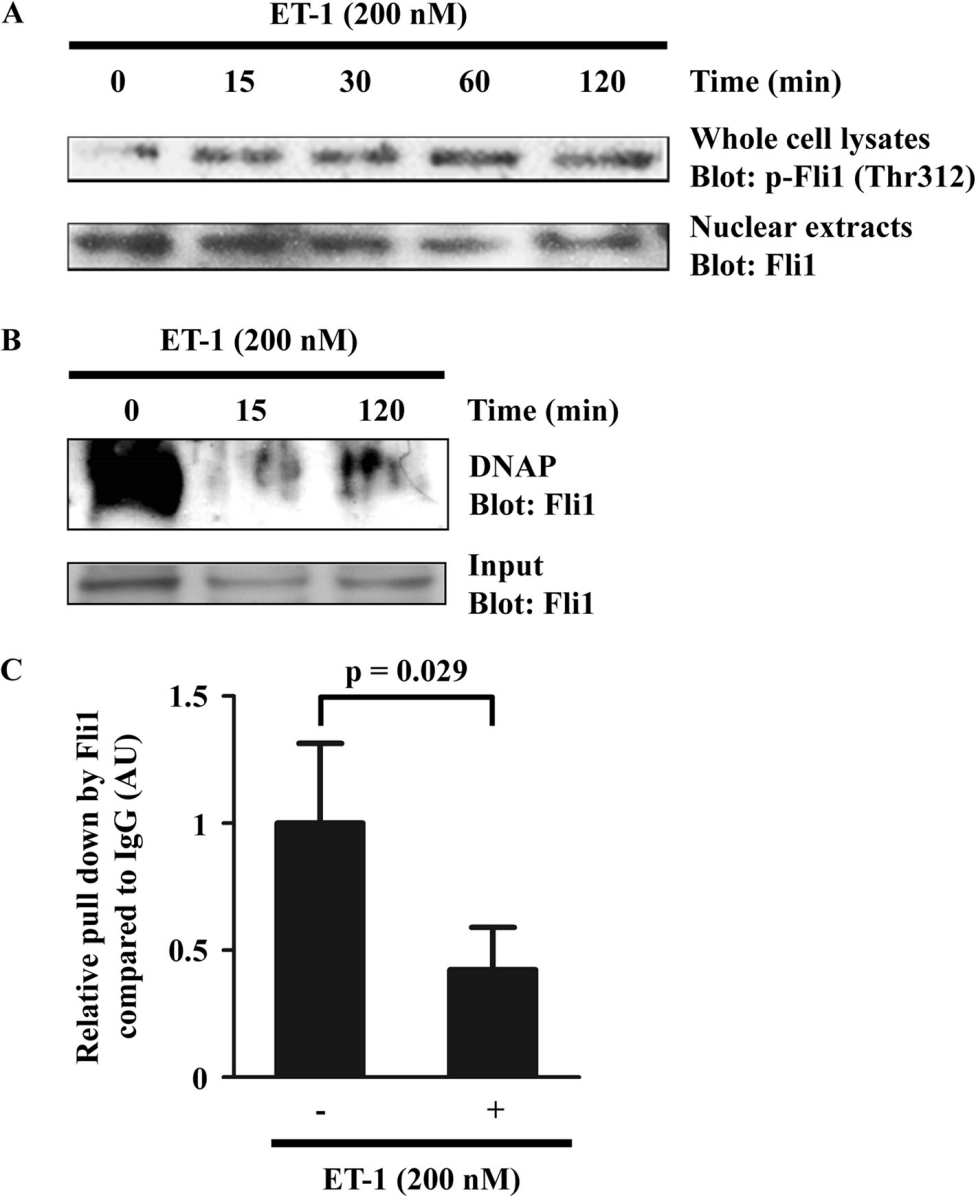
**Endothelin-1 (ET-1) decreased the binding of Fli1 to the human α2 (I) collagen (COL1A2) promoter via increasing its phosphorylation at threonine 312. (A)** Normal dermal fibroblasts were treated with ET-1 for the indicated period of time. Whole cell lysates were subjected to immunoblotting with anti-p-Fli1 antibody. To determine the total Fli1 levels, nuclear extracts were used for immunoblotting with anti-Fli1 antibody. **(B)** Nuclear extracts were incubated with biotin-labeled oligonucleotides. Proteins bound to these nucleotides were isolated with streptavidin-agarose beads, and Fli1 was detected by immunoblotting. Total Fli1 protein levels were determined by immunoblotting using the same nuclear extracts. **(C)** Chromatin was isolated from normal dermal fibroblasts and immunoprecipitated using rabbit anti-Fli1 antibody or rabbit IgG. After isolation of bound DNA, PCR amplification was carried out using COL1A2 promoter-specific primers. Input DNA was taken from each sample before addition of an antibody. The cycle threshold (Ct) values from IgG and Fli1 pull-down were subtracted by the number obtained from 10 × diluted input DNA. The relative pull-down of Fli1 was then normalized by the subtracted Ct value of IgG. The mean value of normal dermal fibroblasts without ET-1 stimulation was arbitrarily set at 1. All bands show one representative of three independent experiments. DNAP, DNA affinity precipitation; AU, arbitrary unit.

To confirm the hypothesis described above, we asked if ET-1 regulates the transcriptional activity of Fli1 in normal dermal fibroblasts. Our previous studies reported that transcriptional activity of Fli1 is tightly regulated by the phosphorylation-acetylation cascade triggered by PKC-δ-dependent phosphorylation of Fli1 at threonine 312 [23]. Phosphorylated Fli1 is subsequently acetylated by p300/CBP-associated factor at lysine 380, resulting in the loss of DNA binding ability [29] and degradation through the proteasomal pathway (unpublished data). As the phosphorylation of Fli1 at threonine 312 is a critical step regulating Fli1 transcriptional activity, we initially looked at the effect of ET-1 on the phosphorylation levels of Fli1 at threonine 312. As shown in Figure 3A, consistent with our hypothesis, Fli1 was phosphorylated at threonine 312 by ET-1 as early as 15 minutes after the stimulation. To further confirm if ET-1 decreases the binding ability of Fli1 to COL1A2 promoter, we employed the DNA affinity precipitation assay. As shown in Figure 3B, ET-1 stimulation decreased the DNA binding ability of Fli1 as early as 15 minutes, consistent with the results of immunoblotting. This observation was also confirmed *in vivo* by ChIP analysis showing that ET-1 decreased the association of Fli1 with the COL1A2 promoter at 3 hours after stimulation (Figure 3C). Taken together, these results indicate that ET-1 increases the promoter activity of the *COL1A2* gene by attenuating the DNA binding of Fli1 through its phosphorylation at threonine 312.

### ET-1 promoted the nuclear localization of PKC-δ by activating c-Abl

**Figure 4:**
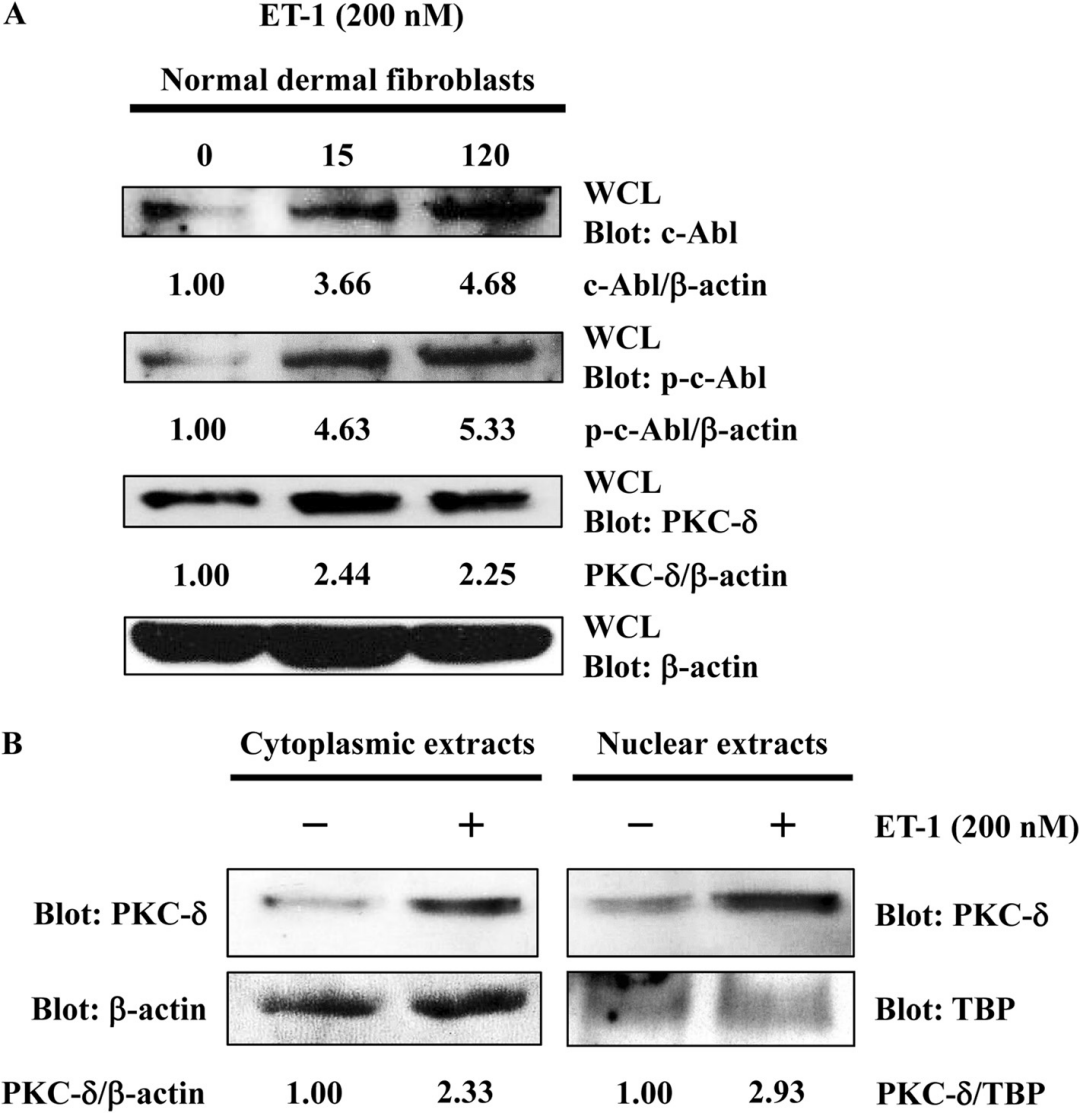
**Endothelin-1 (ET-1) promoted the nuclear localization of protein kinase C (PKC)-δ by activating c-Abl. (A)** Normal dermal fibroblasts were treated with 200 nM of ET-1 for the indicated period of time. The protein levels of c-Abl, PKC-δ, and β-actin and the phosphorylation levels of c-Abl were determined by immunoblotting using whole cell lysates (WCL). **(B)** Normal dermal fibroblasts were treated with ET-1 for 30 minutes and cytoplasmic and nuclear extracts were prepared. The protein levels of PKC-δ in the cytoplasm and nucleus were determined by immunoblotting. Equal amounts of loading were confirmed by immunoblotting for β-actin in cytoplasmic extracts and for TATA binding protein (TBP) in nuclear extracts. The values below each blot represent the relative levels of target molecules normalized by loading controls with densitometry.

We previously disclosed that sequential activation of c-Abl and PKC-δ is necessary for Fli1 phosphorylation at threonine 312 in response to TGF-β stimulation [33]. Therefore, we next examined if ET-1 stimulation sequentially activates c-Abl and PKC-δ in normal dermal fibroblasts. As TGF-β1 increases the expression levels of c-Abl and PKC-δ in normal dermal fibroblasts [34,35], we looked at the effect of ET-1 on the expression levels of c-Abl and PKC-δ by immunoblotting. As shown in Figure 4A, the expression levels of c-Abl and PKC-δ were markedly increased as early as 15 minutes after the ET-1 stimulation in normal dermal fibroblasts. Given that the phosphorylation levels of c-Abl reflect its activation status and nuclear localization is required for PKC-δ to directly phosphorylate Fli1, we also evaluated the phosphorylation levels of c-Abl and nuclear localization of PKC-δ under the same condition. Consistently, ET-1 increased the phosphorylation levels of c-Abl (Figure 4A) and promoted the nuclear translocation of PKC-δ (Figure 4B) in normal dermal fibroblasts. Collectively, these results indicate that ET-1 stimulation activates the c-Abl/PKC-δ/Fli1 pathway and induces the expression of the *COL1A2* gene.

### Bosentan, a dual endothelin receptor antagonist, decreased the expression of the *COL1A2* gene by reversing the transcriptional activity of Fli1 in SSc dermal fibroblasts

Previous reports demonstrated that bosentan, a dual ET receptor antagonist, reverses a pro-fibrotic phenotype of SSc fibroblasts [8]. However, the detailed mechanism by which bosentan exerts its prominent anti-fibrotic effect on SSc fibroblasts has still remained unknown. We previously demonstrated that Fli1 deficiency contributes to the establishment of the pro-fibrotic phenotype in SSc fibroblasts and imatinib mesylate, which targets the c-Abl/PKC-δ/Fli1 pathway, reverses the pro-fibrotic phenotype of these cells [33,36]. Given that SSc fibroblasts are constitutively activated by autocrine stimulation of transforming growth factor- β (TGF-β), a potent inducer of ET-1, and produces an excessive amount of ET-1 [4,9,31,37,38], autocrine ET-1 appears to be involved in the self-activation system in SSc fibroblasts. The present observation that ET-1 inactivates the transcriptional activity of Fli1 suggests that the blockade of autocrine ET-1 by bosentan reverses the pro-fibrotic phenotype of SSc fibroblasts by reactivating the transcriptional repressor activity of Fli1. To address this issue, we performed a series of experiments using cultured SSc dermal fibroblasts.

**Figure 5:**
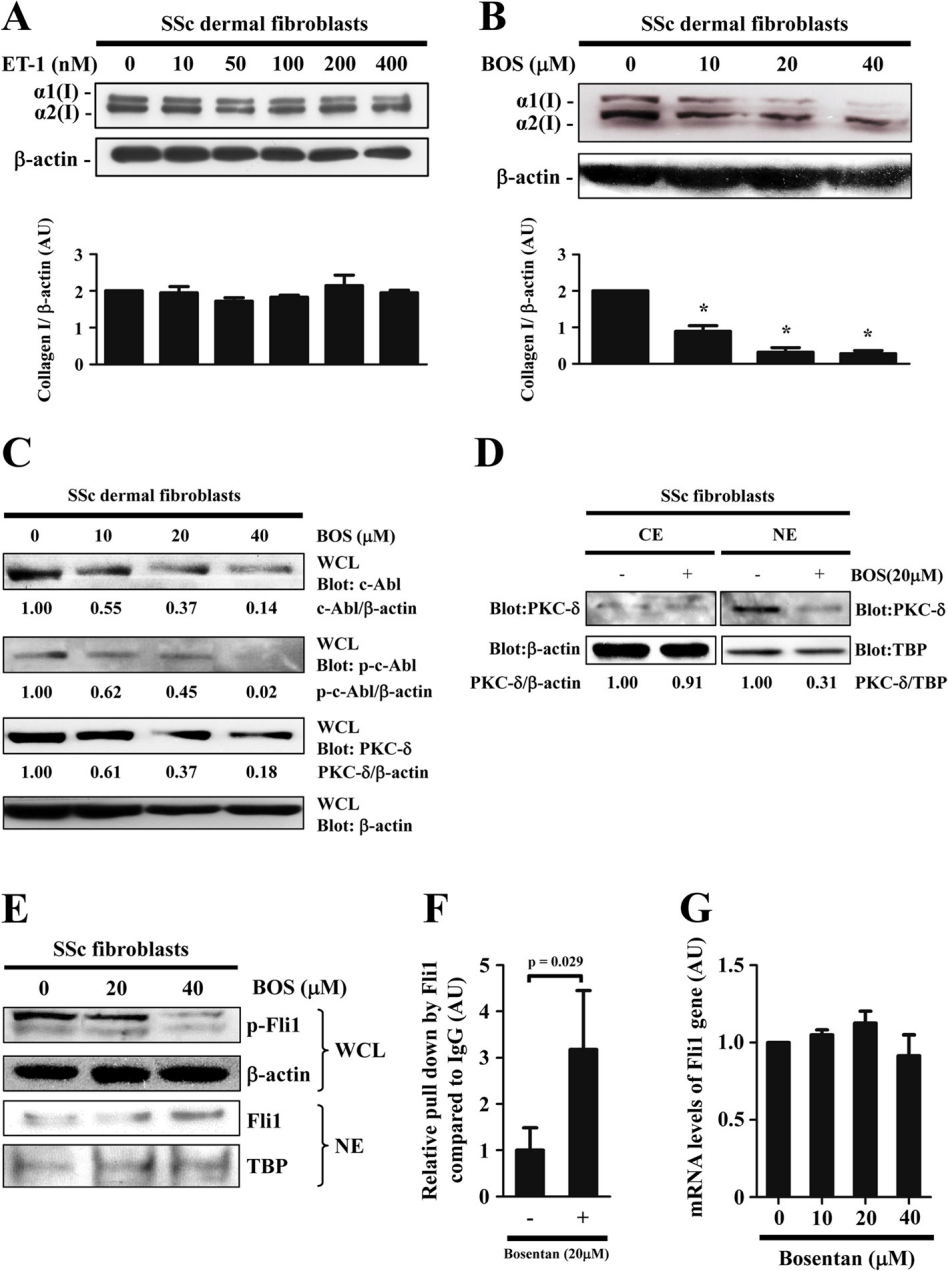
**Bosentan reversed the pro-fibrotic phenotype of systemic sclerosis (SSc) dermal fibroblasts via increasing the DNA binding of Fli1. (A-C)** Whole cell lysates (WCL) prepared from SSc fibroblasts treated with endothelin-1 (ET-1) for 24 hours **(A)** or bosentan (BOS) for 48 hours **(B, C)** were subjected to immunoblotting **(D)** Protein kinase C (PKC)-δ levels were examined by immunoblotting in cytoplasmic extracts (CE) and nuclear extracts (NE) prepared from SSc fibroblasts treated or untreated with bosentan for 48 hours. **(E-G)** SSc fibroblasts were treated or untreated with bosentan for 48 hours. Fli1 phosphorylation levels and total Fli1 levels were determined by immunoblotting **(E)**. Fli1 occupancy of the human α2 (I) collagen (COL1A2) promoter was determined by chromatin immunoprecipitation and reverse transcription (RT) real-time PCR **(F)**. Fli1 mRNA levels were determined by RT real-time PCR **(G)**. The graph represents fold change in type I collagen protein levels quantified by densitometry **(A, B)**, Fli1 occupancy of the COL1A2 promoter **(F)**, and Fli1 mRNA levels **(G)** in comparison to unstimulated controls, which were arbitrarily set at 1. Equal amounts of loading were confirmed by immunoblotting for β-actin in cytoplasmic extracts and for TATA binding protein (TBP) in nuclear extracts. The values below each blot represent the relative levels of target molecules normalized by loading controls with densitometry. α1(I), α1(I) procollagen; α2(I), α2(I) procollagen; AU, arbitrary unit.

Supporting the contribution of autocrine ET-1 to the activation of SSc dermal fibroblasts, exogenous ET-1 did not affect type I collagen expression (Figure 5A), whereas bosentan suppressed the expression of type I collagen in a dose-dependent manner without any effect on cell viability in SSc dermal fibroblasts (Figure 5B and Table 1). Furthermore, the total levels and the phosphorylation levels of c-Abl and the total levels and nuclear localization of PKC-δ were decreased in SSc dermal fibroblasts treated with bosentan (Figure 5C and 5D). Consistently, Fli1 phosphorylation at threonine 312 was reduced (Figure 5E) and the occupancy of Fli1 on COL1A2 promoter was increased in SSc dermal fibroblasts treated with bosentan (Figure 5F). Importantly, bosentan did not affect the mRNA levels of the *FLI1* gene in SSc dermal fibroblasts (Figure 5G). Collectively, these results indicate that autocrine ET-1 contributes to the activation of SSc dermal fibroblasts and bosentan reverses a pro-fibrotic phenotype of SSc dermal fibroblasts by increasing the DNA binding ability of Fli1.

**Table 1:** Viability of systemic sclerosis dermal fibroblasts in the presence or absence of bosentan, which was evaluated by trypan blue exclusion test

**Duration of treatment (h)**	**Bosentan (μM)**			
	**0**	**10**	**20**	**40**
0	96.5 ± 0.05	98.8 ± 0.24	96.3 ± 0.70	97.3 ± 1.52
24	97.3 ± 1.13	97.8 ± 1.13	97.6 ± 1.68	96.3 ± 0.72
48	95.6 ± 0.64	96.2 ± 0.55	96.8 ± 0.25	95.1 ± 0.18

The impact of bosentan on cell viability was evaluated by trypan blue exclusion test. The percentage of cell viability was calculated according to the following formula: % cell viability = (viable cell count/total cell count) × 100. Data are expressed as the mean ± SD of results in three independent experiments.

### Bosentan increased the expression of Fli1 protein in lesional dermal fibroblasts of the BLM-induced murine model of SSc

**Figure 6:**
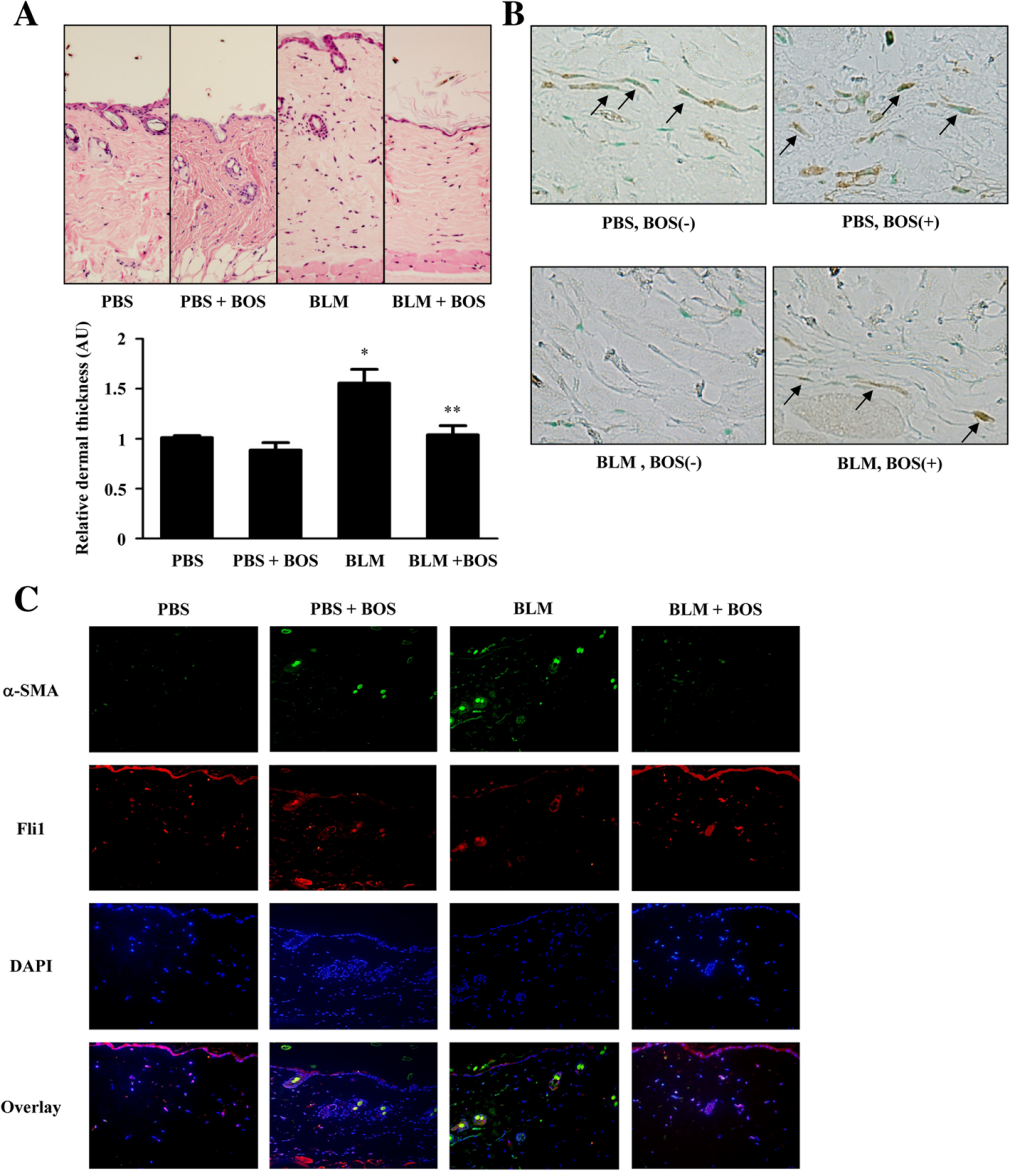
**Bosentan prevented the development of dermal fibrosis, at least partially, by increasing the expression of Fli1 protein in lesional dermal fibroblasts of bleomycin (BLM)-induced systemic sclerosis (SSc) murine model.** Wild-type C57BL/6 mice were injected with BLM (200 μg) or PBS into the skin of the back for 3 weeks. In some mice, bosentan (BOS) was administered intraperitoneally for 4 weeks from 1 week before BLM treatment. **(A)** The thickness of the skin on the back was evaluated by hematoxylin and eosin staining. The graph represents fold change in the dermal thickness in comparison to PBS-treated mice without bosentan administration, which was arbitrarily set at 1. **(B)** The expression levels of Fli1 protein were determined by immunohistochemistry. The arrows represent Fli1-positive dermal fibroblasts. **(C)** Double immunofluorescence for Fli1 (red) and α-SMA (green). Blue signals of nuclei were detected with 4′,6-diamidino-2-phenylindole (DAPI). The bottom panels represent the overlay of the three above. *P <0.05 versus PBS-treated mice without bosentan. **P <0.05 versus BLM-treated mice without bosentan. AU, arbitrary unit.

Finally, we investigated if bosentan increases the expression of Fli1 protein in lesional dermal fibroblasts of the BLM-induced SSc murine model because previous reports demonstrated that bosentan prevents the development of dermal fibrosis in this model [12]. As we could reproduce the preventive effect of bosentan on dermal fibrosis in BLM-treated mice (Figure 6A), we carried out immunostaining for Fli1 in the skin samples taken from these mice. As shown in Figure 6B, in the absence of bosentan, the number of Fli1-positive dermal fibroblasts was much more decreased in dermal fibroblasts of BLM-treated mice than in those of PBS-treated mice. In contrast, when administered bosentan, the number of Fli1-positive dermal fibroblasts was comparable between BLM-treated mice and PBS-treated mice. Importantly, the signals of Fli1 and α-SMA, a marker of myofibroblasts, in double immunofluorescence were mutually exclusive in most of dermal fibroblasts (Figure 6C), indicating that Fli1 expression is closely related to the inactivation of dermal fibroblasts *in vivo*. Collectively, these results suggest that bosentan prevents the development of dermal fibrosis in the BLM-induced SSc murine model, at least partially, by increasing the expression of Fli1 protein in lesional dermal fibroblasts.

## Discussion

This study was undertaken to clarify the molecular mechanism underlying the pro-fibrotic effect of ET-1 on normal dermal fibroblasts and anti-fibrotic effect of bosentan on SSc dermal fibroblasts. A series of experiments demonstrated that ET-1 activates the c-Abl/PKC-δ/Fli1 pathway and reduces the DNA binding ability of Fli1, resulting in the induction of type I collagen expression. Given that a pro-fibrotic phenotype of SSc fibroblasts is largely due to stimulation by autocrine TGF-β, a potent inducer of ET-1, and those cells produce a much larger amount of ET-1 than normal dermal fibroblasts [1,7,38], it was speculated that the blockade of ET-1-dependent signaling by bosentan reverses the pro-fibrotic phenotype of SSc dermal fibroblasts by increasing the DNA binding of Fli1 through the inhibition of the c-Abl /PKC-δ/Fli1 pathway, which is constitutively activated in those cells [33]. Supporting this hypothesis, exogenous ET-1 did not affect type I collagen expression, whereas bosentan suppressed the expression of c-Abl and PKC-δ, decreased the nuclear localization of PKC-δ and Fli1 phosphorylation at threonine 312, and eventually increased the DNA binding ability of Fli1 to the COL1A2 promoter resulting in the reduction of type I collagen expression in SSc dermal fibroblasts. Furthermore, bosentan increased the expression levels of Fli1 protein in lesional dermal fibroblasts of BLM-treated mice. As the protein stability of Fli1 increases when Fli1 binds to DNA, and is protected from degradation, these *in vivo* data suggest that bosentan prevents the development of dermal fibrosis in BLM-treated mice by increasing the DNA binding of Fli1 in lesional dermal fibroblasts. Collectively, these results indicate that the anti-fibrotic effect of bosentan on SSc dermal fibroblasts and BLM-induced SSc murine model is at least partly attributable to the increase in the DNA binding ability of Fli1.

ET-1 induces a pro-fibrotic phenotype in fibroblasts through increasing the expression of extracellular matrix (ECM) proteins, such as type I and III collagen and fibronectin, and decreasing the expression of matrix metalloproteinase 1 [39,40], therefore the blockade of ET-1 signaling has been thought to be a potential therapeutic strategy for fibrotic disorders, including SSc. The first study of the anti-fibrotic effect of bosentan on SSc fibroblasts was reported in 2004 by Shi-Wen *et al*. A series of studies from their group demonstrated that bosentan suppresses the expression of α-SMA, type I collagen, fibronectin, and CCN2 in SSc lung fibroblasts and ET-1 is a downstream mediator of pro-fibrotic responses to TGF-β in human lung fibroblasts [8,10,11]. Following these, Lagares *et al*. [12] revealed that these previous findings in lung fibroblasts are reproducible in dermal fibroblasts in *in vitro* experiments and *in vivo* animal models. Thus, ET-1 and its receptor antagonists have currently drawn much attention in the field of research on the mechanism and treatment of fibrotic disorders, but the detailed molecular mechanism explaining their effects has remained to be clarified. Since ET-1 induces a prominent fibrotic response even in the absence of Smad2/3 activation, which are important intracellular second messengers of TGF-β signaling especially in the regulation of the fibrotic gene program, ET-1 appears to inactivate a potent master repressor acting on a set of fibrosis-related genes. Taking into account that gene silencing of Fli1 results in the induction of *COL1A2* gene expression up to the level much greater than that achieved by TGF-β1 stimulation [23,25,29,32,33,41], we speculated that Fli1 may be inactivated in response to ET-1 stimulation. Consistent with this idea, a responsive element of COL1A2 promoter to ET-1 was located between −353 and −264 bp, including the Fli1 binding site, and ET-1 activated c-Abl and PKC-δ, resulting in Fli1 inactivation through its phosphorylation at threonine 312. Although other mechanisms may be involved in the pro-fibrotic effect of ET-1, the current data indicate that Fli1 largely contributes to the mechanism explaining the potent pro-fibrotic effect of ET-1 in dermal fibroblasts.

Although bosentan attenuates BLM-induced dermal and pulmonary fibrosis in animal models [12,42-44], its efficacy on skin sclerosis and ILD in SSc, to our best knowledge, has been reported to be limited [20,21]. In addition to the difference in the pathological process between SSc and animal models, lower potential of bosentan for good tissue distribution, which is estimated around 1% [22], has been cited as a cause of the unsuccessful outcome of oral bosentan therapy against skin sclerosis and ILD in SSc. Considering that the average peak plasma concentration after a single dose of 125 mg of bosentan, which is a maximal dose clinically used in humans, is 1,584 ng/ml (2.87 μM) in Caucasian and 1,922 ng/ml (3.48 μM) in Japanese subjects [45], 10 μM of bosentan, which has been used for *in vitro* experiments with fibroblasts, appears to be much higher than the concentration of bosentan in human skin tissue. In the present study, bosentan suppressed type I collagen expression in a dose-dependent manner up to 40 μM without affecting cell viability in SSc dermal fibroblasts, suggesting that endothelin receptor antagonists with better tissue distribution and tolerability may have potential as disease-modifying drugs targeting skin sclerosis and ILD in SSc by directly inactivating fibroblasts.

According to a previous report by Wang *et al*. [46], the expression of Fli1 is strongly suppressed at the transcription level by an epigenetic mechanism in SSc dermal fibroblasts. The authors demonstrated that the acetylation levels of histone H3 and H4 are decreased, while the methylation levels of CpG islands are increased, in the promoter region of the *FLI1* gene in SSc dermal fibroblasts compared with normal dermal fibroblasts, suggesting that the decrease in *FLI1* gene expression contributes to the developmental process of SSc as a potential predisposing factor. The most important observation in the present study was that bosentan reversed the decreased expression of Fli1 protein without affecting its gene expression status in SSc dermal fibroblasts. These results suggest that bosentan can increase Fli1 protein levels regardless of the degree of epigenetic transcriptional suppression in those cells. Given that the reversion of epigenetically repressed disease-related genes by drugs is a general idea for the treatment of various diseases, bosentan may serve as a disease-modifying drug for SSc. Although the efficacy of bosentan on skin sclerosis and ILD is limited, recent papers demonstrated the potential of bosentan as a disease-modifying drug for SSc vasculopathy. In addition to the prevention of new digital ulcers by bosentan [15,16], Guiducci *et al*. [47] revealed that one-year treatment with bosentan significantly decreases the number of advanced nailfold capillary changes, such as capillary disorganization, ramified capillaries, and capillary loss, while increasing the number of relatively early changes, including enlarged capillaries, megacapillaries, and hemorrhage, in SSc patients. These clinical findings suggest that the effect of endothelin receptor antagonists on SSc vasculopathy goes beyond the reversal of the potent vasoconstrictive effects of endothelin. This notion has been supported by some experimental data. For example, ET-1 has a potent mitogenic action on fibroblasts and vascular smooth muscle cells [48,49]. Furthermore, ET-1 prolongs the survival of myofibroblasts by preventing apoptosis [8]. Moreover, ET-1 triggers the pathological inflammation by modulating the expression of cell adhesion molecules on endothelial cells [50] and by promoting the production of free radical and fibrosis-inducing cytokines, such as monocyte chemoattractant protein-1 and TGF-β, from macrophages [51]. These proliferative, pro-fibrotic, and pro-inflammatory effects of ET-1 contribute to the development of SSc vasculopathy. Considering that endothelial Fli1 deficiency contributes to the development of SSc vasculopathy [52], bosentan may exert its disease-modifying effect on SSc vasculopathy by increasing endothelial Fli1 expression. The research regarding the effect of bosentan on endothelial Fli1 deficiency is currently underway in our laboratory.

Similar to bosentan, our latest paper demonstrated that imatinib reverses the decreased expression of Fli1 in SSc dermal fibroblasts by increasing its protein stability and without affecting its gene expression status [33], suggesting that imatinib is a potential disease-modifying drug for SSc patients. Consistent with this idea, imatinib moderately improved skin sclerosis of SSc patients in a couple of case reports [53,54] and case series [55-57]. Although imatinib was poorly tolerated and failed to show a significant effect on skin sclerosis of SSc patients in a couple of clinical trials [58], previous case reports suggest that some derivatives of imatinib with a better tolerability may be effective for skin sclerosis in a certain subset of SSc patients. Similarly, the clinical response of digital ulcers associated with SSc to bosentan varies from patients to patients [15,16]. Given that SSc is a multifactorial disease caused by a complex interaction between hereditary factors and environmental influences, these clinical data suggest that imatinib and bosentan may target some disease-associated factors, leading to the modification of the natural disease course in a certain subset of SSc patients. Given that both of c-Abl tyrosine kinase inhibitors and endothelin receptor antagonists target Fli1, a potential predisposing factor of SSc, further studies on the association between the degree of Fli1 suppression and the clinical efficacy of these drugs may provide us some clue to properly select good responders and effectively administer these treatments for SSc patients.

In the present study, 200 nM of ET-1 showed the maximal stimulatory effect on type I collagen expression in normal human dermal fibroblasts. However, previous reports demonstrated that 100 nM of ET-1 exhibits a significant stimulatory effect on the expression levels of type I collagen, fibronectin, fibrillin-1, and α-SMA and the gel contraction in murine embryonic fibroblasts and human normal lung and dermal fibroblasts [8,9,12,40,59]. This discrepancy may be attributable to the different sensitivity of fibroblasts to ET-1 and/or to the different experimental conditions between each study, but the actual reason is unclear.

## Conclusions

We herein reported the first study regarding the detailed molecular mechanism underlying the pro-fibrotic effect of ET-1 on normal dermal fibroblasts and the anti-fibrotic effect of bosentan on SSc dermal fibroblasts, in which transcription factor Fli1, a potential predisposing factor in SSc, is an important target. Although the efficacy of bosentan for dermal and pulmonary fibrotic conditions associated with SSc is limited, the present observation definitely provides us with a useful clue to further explore the potential of the upcoming new dual endothelin receptor antagonists with better clinical effects as disease-modifying drugs for SSc.

## Abbreviations

β-GAL: β-galactosidase; BLM: bleomycin; bp: base pairs; CAT: chloramphenicol acetyltransferase; ChIP: Chromatin immunoprecipitation; COL1A2: human α2 (I) collagen; ET-1: endothelin-1; EBS: Ets binding site; ILD: interstitial lung disease; PBS: phosphate buffered saline; PKC: protein kinase C; RT: reverse transcription; SSc: systemic sclerosis; TGF: transforming growth factor.

## Competing interests

Y Asano has received honoraria and research funding from Acterion.

## Authors’ contributions

KA: data collection and analysis, drafting the manuscript and final approval of the manuscript. YA: conception and design, analysis and interpretation of data, manuscript writing, critical revision and final approval of the manuscript. NA: data collection and analysis, drafting the manuscript and final approval of the manuscript. SN: data collection and analysis, drafting the manuscript and final approval of the manuscript. T Taniguchi: data collection and analysis, drafting the manuscript and final approval of the manuscript. T Takahashi: data collection and analysis, drafting the manuscript and final approval of the manuscript. YI: data collection and analysis, drafting the manuscript and final approval of the manuscript. T Toyama: data collection and analysis, drafting the manuscript and final approval of the manuscript. SS: analysis and interpretation of data, critical revision and final approval of the manuscript. All authors read and approved the final manuscript.

## References

[B1] Asano Y Future treatments in systemic sclerosis J Dermatol 2010 37 54 70 10.1111/j.1346-8138.2009.00758.x 20175840

[B2] Asano Y Ihn H Yamane K Kubo M Tamaki K Increased expression levels of integrin αVβ5 on scleroderma fibroblasts Am J Pathol 2004 164 1275 1292 10.1016/S0002-9440(10)63215-4 15039216 PMC1615355

[B3] Asano Y Ihn H Yamane K Jinnin M Mimura Y Tamaki K Increased expression of integrin αVβ3 contributes to the establishment of autocrine TGF-β signaling in scleroderma fibroblasts J Immunol 2005 175 7708 7718 16301681 10.4049/jimmunol.175.11.7708

[B4] Asano Y Ihn H Yamane K Jinnin M Mimura Y Tamaki K Involvement of αVβ5 integrin-mediated activation of latent transforming growth factor β1 in autocrine transforming growth factor β signaling in systemic sclerosis fibroblasts Arthritis Rheum 2005 52 2897 2905 10.1002/art.21246 16142753

[B5] Asano Y Ihn H Yamane K Jinnin M Tamaki K Increased expression of integrin αVβ5 induces the myofibroblastic differentiation of dermal fibroblasts Am J Pathol 2006 168 499 510 10.2353/ajpath.2006.041306 16436664 PMC1606497

[B6] Mimura Y Ihn H Jinnin M Asano Y Yamane K Tamaki K Constitutive thrombospondin-1 overexpression contributes to autocrine transforming growth factor-β signaling in cultured scleroderma fibroblasts Am J Pathol 2005 166 1451 1463 10.1016/S0002-9440(10)62362-0 15855645 PMC1606399

[B7] Varga J Pasche B Transforming growth factor β as a therapeutic target in systemic sclerosis Nat Rev Rheumatol 2009 5 200 206 10.1038/nrrheum.2009.26 19337284 PMC3959159

[B8] Shi-Wen X Chen Y Denton CP Eastwood M Renzoni EA Bou-Gharios G Pearson JD Dashwood M du Bois RM Black CM Leask A Abraham DJ Endothelin-1 promotes myofibroblast induction through the ETA receptor via a rac/phosphoinositide 3-kinase/Akt-dependent pathway and is essential for the enhanced contractile phenotype of fibrotic fibroblasts Mol Biol Cell 2004 15 2707 2719 10.1091/mbc.E03-12-0902 15047866 PMC420095

[B9] Shi-Wen X Rodríguez-Pascual F Lamas S Holmes A Howat S Pearson JD Dashwood MR du Bois RM Denton CP Black CM Abraham DJ Leask A Constitutive ALK5-independent c-Jun N-terminal kinase activation contributes to endothelin-1 overexpression in pulmonary fibrosis: evidence of an autocrine endothelin loop operating through the endothelin A and B receptors Mol Cell Biol 2006 26 5518 5527 10.1128/MCB.00625-06 16809784 PMC1592704

[B10] Shi-Wen X Renzoni EA Kennedy L Howat S Chen Y Pearson JD Bou-Gharios G Dashwood MR du Bois RM Black CM Denton CP Abraham DJ Leask A Endogenous endothelin-1 signaling contributes to type I collagen and CCN2 overexpression in fibrotic fibroblasts Matrix Biol 2007 26 625 632 10.1016/j.matbio.2007.06.003 17681742

[B11] Shi-wen X Kennedy L Renzoni EA Bou-Gharios G du Bois RM Black CM Denton CP Abraham DJ Leask A Endothelin is a downstream mediator of profibrotic responses to transforming growth factor b in human lung fibroblasts Arthritis Rheum 2007 56 4189 4194 10.1002/art.23134 18050250

[B12] Lagares D García-Fernández RA Jiménez CL Magán-Marchal N Busnadiego O Lamas S Rodríguez-Pascual F Endothelin 1 contributes to the effect of transforming growth factor b1 on wound repair and skin fibrosis Arthritis Rheum 2010 62 878 889 10.1002/art.27307 20131241

[B13] Yamane K Miyauchi T Suzuki N Yuhara T Akama T Suzuki H Kashiwagi H Significance of plasma endothelin-1 levels in patients with systemic sclerosis J Rheumatol 1992 19 1566 1571 1464869

[B14] Vancheeswaran R Magoulas T Efrat G Wheeler-Jones C Olsen I Penny R Black CM Circulating endothelin-1 levels in systemic sclerosis subsets--a marker of fibrosis or vascular dysfunction? J Rheumatol 1994 21 1838 1844 7837147

[B15] Korn JH Mayes M Matucci Cerinic M Rainisio M Pope J Hachulla E Rich E Carpentier P Molitor J Seibold JR Hsu V Guillevin L Chatterjee S Peter HH Coppock J Herrick A Merkel PA Simms R Denton CP Frust D Nguyen N Gaitonde M Black C Digital ulcers in systemic sclerosis: prevention by treatment with bosentan, an oral endothelin receptor antagonist Arthritis Rheum 2004 50 3985 3993 10.1002/art.20676 15593188

[B16] Matucci-Cerinic M Denton CP Furst DE Mayes MD Hsu VM Carpentier P Wigley FM Black CM Fessler BJ Merkel PA Pope JE Sweiss NJ Doyle MK Hellmich B Medsger TA Jr Morganti A Kramer F Korn JH Seibold JR Bosentan treatment of digital ulcers related to systemic sclerosis: results from the RAPIDS-2 randomised, double-blind, placebo-controlled trial Ann Rheum Dis 2011 70 32 38 20805294 10.1136/ard.2010.130658 PMC3002766

[B17] Denton CP Humbert M Rubin L Black CM Bosentan treatment for pulmonary arterial hypertension related to connective tissue disease: a subgroup analysis of the pivotal clinical trials and their open-label extensions Ann Rheum Dis 2006 65 1336 1340 16793845 10.1136/ard.2005.048967 PMC1798307

[B18] McLaughlin VV Survival in patients with pulmonary arterial hypertension treated with first-line bosentan Eur J Clin Invest 2006 36 10 15 10.1111/j.1365-2362.2006.01688.x 16919005

[B19] Williams MH Das C Handler CE Akram MR Davar J Denton CP Smith CJ Black CM Coghlan JG Systemic sclerosis associated pulmonary hypertension: improved survival in the current era Heart 2006 92 926 932 10.1136/hrt.2005.069484 16339813 PMC1860719

[B20] Seibold JR Denton CP Furst DE Guillevin L Rubin LJ Wells A Matucci Cerinic M Riemekasten G Emery P Chadha-Boreham H Charef P Roux S Black CM Randomized, prospective, placebo-controlled trial of bosentan in interstitial lung disease secondary to systemic sclerosis Arthritis Rheum 2010 62 2101 2108 20506355 10.1002/art.27466

[B21] Furuya Y Kuwana M Effect of Bosentan on systemic sclerosis-associated interstitial lung disease ineligible for cyclophosphamide therapy: a prospective open-label study J Rheumatol 2011 38 2186 2192 10.3899/jrheum.110499 21885489

[B22] Iglarz M Binkert C Morrison K Fischli W Gatfield J Treiber A Weller T Bolli MH Boss C Buchmann S Capeleto B Hess P Qiu C Clozel M Pharmacology of macitentan, an orally active tissue-targeting dual endothelin receptor antagonist J Pharmacol Exp Ther 2008 327 736 745 10.1124/jpet.108.142976 18780830

[B23] Asano Y Trojanowska M Phosphorylation of Fli1 at threonine 312 by protein kinase C δ promotes its interaction with p300/CREB-binding protein-associated factor and subsequent acetylation in response to transforming growth factor β Mol Cell Biol 2009 29 1882 1894 10.1128/MCB.01320-08 19158279 PMC2655609

[B24] Ihn H Tamaki K Competition analysis of the human α2(I) collagen promoter using synthetic oligonucleotides J Invest Dermatol 2000 114 1011 1016 10.1046/j.1523-1747.2000.00956.x 10771485

[B25] Nakerakanti SS Kapanadze B Yamasaki M Markiewicz M Trojanowska M Fli1 and Ets1 have distinct roles in connective tissue growth factor/CCN2 gene regulation and induction of the profibrotic gene program J Biol Chem 2006 281 25259 25269 10.1074/jbc.M600466200 16829517

[B26] Akada H Yan D Zou H Fiering S Hutchison RE Mohi MG Conditional expression of heterozygous or homozygous Jak2V617F from its endogenous promoter induces a polycythemia vera-like disease Blood 2010 115 3589 3597 10.1182/blood-2009-04-215848 20197548 PMC2867267

[B27] Tamaki T Ohnishi K Hartl C LeRoy EC Trojanowska M Characterization of a GC-rich region containing Sp1 binding site (s) as a constitutive responsive element of the α2(I) collagen gene in human fibroblasts J Biol Chem 1995 270 4299 4304 10.1074/jbc.270.9.4299 7876190

[B28] Boast S Su MW Ramirez F Sanchez M Avvedimento EV Functional analysis of cis-acting DNA sequences controlling transcription of the human type I collagen genes J Biol Chem 1990 265 13351 13356 2376598

[B29] Asano Y Czuwara J Trojanowska M Transforming growth factor-β regulates DNA binding activity of transcription factor Fli1 by p300/CREB-binding protein-associated factor-dependent acetylation J Biol Chem 2007 282 34672 34683 10.1074/jbc.M703907200 17884818

[B30] Yoshizaki A Iwata Y Komura K Ogawa F Hara T Muroi E Takenaka M Shimizu K Hasegawa M Fujimoto M Tedder TF Sato S CD19 regulates skin and lung fibrosis via Toll-like receptor signaling in a model of bleomycin-induced scleroderma Am J Pathol 2008 172 1650 1663 10.2353/ajpath.2008.071049 18467694 PMC2408424

[B31] Asano Y Ihn H Yamane K Jinnin M Mimura Y Tamaki K Phosphatidylinositol 3-kinase is involved in α2(I) collagen gene expression in normal and scleroderma fibroblasts J Immunol 2004 172 7123 7135 15153536 10.4049/jimmunol.172.11.7123

[B32] Czuwara-Ladykowska J Shirasaki F Jackers P Watson DK Trojanowska M Fli-1 inhibits collagen type I production in dermal fibroblasts via an Sp1-dependent pathway J Biol Chem 2001 276 20839 20848 10.1074/jbc.M010133200 11278621

[B33] Bujor AM Asano Y Haines P Lafyatis R Trojanowska M The c-Abl tyrosine kinase controls protein kinase Cδ-induced Fli-1 phosphorylation in human dermal fibroblasts Arthritis Rheum 2011 63 1729 1737 21321929 10.1002/art.30284 PMC3381734

[B34] Bhattacharyya S Ishida W Wu M Wilkes M Mori Y Hinchcliff M Leof E Varga J A non-Smad mechanism of fibroblast activation by transforming growth factor-β via c-Abl and Egr-1: selective modulation by imatinib mesylate Oncogene 2009 28 1285 1297 10.1038/onc.2008.479 19151753 PMC4006376

[B35] Jinnin M Ihn H Yamane K Mimura Y Asano Y Tamaki K α2(I) collagen gene regulation by protein kinase C signaling in human dermal fibroblasts Nucleic Acids Res 2005 33 1337 1351 10.1093/nar/gki275 15741186 PMC552962

[B36] Kubo M Czuwara-Ladykowska J Moussa O Markiewicz M Smith E Silver RM Jablonska S Blaszczyk M Watson DK Trojanowska M Persistent down-regulation of Fli1, a suppressor of collagen transcription, in fibrotic scleroderma skin Am J Pathol 2003 163 571 581 10.1016/S0002-9440(10)63685-1 12875977 PMC1868228

[B37] Asano Y Ihn H Yamane K Kubo M Tamaki K Impaired Smad7-Smurf-mediated negative regulation of TGF-β signaling in scleroderma fibroblasts J Clin Invest 2004 113 253 264 10.1172/JCI16269 14722617 PMC310747

[B38] Kawaguchi Y Suzuki K Hara M Hidaka T Ishizuka T Kawagoe M Nakamura H Increased endothelin-1 production in fibroblasts derived from patients with systemic sclerosis Ann Rheum Dis 1994 53 506 510 7944634 10.1136/ard.53.8.506 PMC1005389

[B39] Shi-Wen X Denton CP Dashwood MR Holmes AM Bou-Gharios G Pearson JD Black CM Abraham DJ Fibroblast matrix gene expression and connective tissue remodeling: role of endothelin-1 J Invest Dermatol 2001 116 417 425 10.1046/j.1523-1747.2001.01256.x 11231316

[B40] Soldano S Montagna P Villaggio B Parodi A Gianotti G Sulli A Seriolo B Secchi ME Cutolo M Endothelin and sex hormones modulate the fibronectin synthesis by cultured human skin scleroderma fibroblasts Ann Rheum Dis 2009 68 599 602 18952637 10.1136/ard.2008.097378 PMC2651484

[B41] Asano Y Markiewicz M Kubo M Szalai G Watson DK Trojanowska M Transcription factor Fli1 regulates collagen fibrillogenesis in mouse skin Mol Cell Biol 2009 29 425 434 10.1128/MCB.01278-08 19001092 PMC2612518

[B42] Schroll S Arzt M Sebah D Nüchterlein M Blumberg F Pfeifer M Improvement of bleomycin-induced pulmonary hypertension and pulmonary fibrosis by the endothelin receptor antagonist Bosentan Respir Physiol Neurobiol 2010 170 32 36 10.1016/j.resp.2009.11.005 19931426

[B43] Mutsaers SE Marshall RP Goldsack NR Laurent GJ McAnulty RJ Effect of endothelin receptor antagonists (BQ-485, Ro 47–0203) on collagen deposition during the development of bleomycin-induced pulmonary fibrosis in rats Pulm Pharmacol Ther 1998 11 221 225 10.1006/pupt.1998.0142 9918760

[B44] Park SH Saleh D Giaid A Michel RP Increased endothelin-1 in bleomycin-induced pulmonary fibrosis and the effect of an endothelin receptor antagonist Am J Respir Crit Care Med 1997 156 600 608 10.1164/ajrccm.156.2.9607123 9279246

[B45] van Giersbergen PL Dingemanse J Comparative investigation of the pharmacokinetics of bosentan in Caucasian and Japanese healthy subjects J Clin Pharmacol 2005 45 42 47 10.1177/0091270004270833 15601804

[B46] Wang Y Fan PS Kahaleh B Association between enhanced type I collagen expression and epigenetic repression of the FLI1 gene in scleroderma fibroblasts Arthritis Rheum 2006 54 2271 2279 10.1002/art.21948 16802366

[B47] Guiducci S Bellando Randone S Bruni C Carnesecchi G Maresta A Iannone F Lapadula G Matucci Cerinic M Bosentan fosters microvascular de-remodelling in systemic sclerosis Clin Rheumatol 2012 31 1723 1725 10.1007/s10067-012-2074-5 23053682

[B48] Cambrey AD Harrison NK Dawes KE Southcott AM Black CM du Bois RM Laurent GJ McAnulty RJ Increased levels of endothelin-1 in bronchoalveolar lavage fluid from patients with systemic sclerosis contribute to fibroblast mitogenic activity in vitro Am J Respir Cell Mol Biol 1994 11 439 445 10.1165/ajrcmb.11.4.7917311 7917311

[B49] Yang Z Krasnici N Luscher TF Endothelin-1 potentiates human smooth muscle cell growth to PDGF: effects of ETA and ETB receptor blockade Circulation 1999 100 5 8 10.1161/01.CIR.100.1.5 10393673

[B50] Iannone F Riccardi MT Guiducci S Bizzoca R Cinelli M Matucci-Cerinic M Lapadula G Bosentan regulates the expression of adhesion molecules on circulating T cells and serum soluble adhesion molecules in systemic sclerosis-associated pulmonary arterial hypertension Ann Rheum Dis 2008 67 1121 1126 18029384 10.1136/ard.2007.080424 PMC2564790

[B51] Abraham D Role of endothelin in lung fibrosis Eur Respir Rev 2008 17 145 150 10.1183/09059180.00010907

[B52] Asano Y Stawski L Hant F Highland K Silver R Szalai G Watson DK Trojanowska M Endothelial Fli1 deficiency impairs vascular homeostasis: a role in scleroderma vasculopathy Am J Pathol 2010 176 1983 1998 10.2353/ajpath.2010.090593 20228226 PMC2843486

[B53] Tamaki Z Asano Y Hatano M Yao A Kawashima T Tomita M Kinugawa K Nagai R Sato S Efficacy of low-dose imatinib mesylate for cutaneous involvement in systemic sclerosis: a preliminary report of three cases Mod Rheumatol 2012 22 94 99 10.3109/s10165-011-0472-1 21633912

[B54] Sfikakis PP Gorgoulis VG Katsiari CG Evangelou K Kostopoulos C Black CM Imatinib for the treatment of refractory, diffuse systemic sclerosis Rheumatology (Oxford) 2008 47 735 737 10.1093/rheumatology/ken104 18326532

[B55] Spiera RF Gordon JK Mersten JN Magro CM Mehta M Wildman HF Kloiber S Kirou KA Lyman S Crow MK Imatinib mesylate (Gleevec) in the treatment of diffuse cutaneous systemic sclerosis: results of a 1-year, phase IIa, single-arm, open-label clinical trial Ann Rheum Dis 2011 70 1003 1009 21398330 10.1136/ard.2010.143974 PMC3086082

[B56] Khanna D Saggar R Mayes MD Abtin F Clements PJ Maranian P Assassi S Singh RR Furst DE A one-year, phase I/IIa, open-label pilot trial of imatinib mesylate in the treatment of systemic sclerosis-associated active interstitial lung disease Arthritis Rheum 2011 63 3540 3546 10.1002/art.30548 21769849 PMC3205223

[B57] Guo L Chen XX Gu YY Zou HJ Ye S Low-dose imatinib in the treatment of severe systemic sclerosis: a case series of six Chinese patients and literature review Clin Rheumatol 2012 31 1395 1400 10.1007/s10067-012-2032-2 22875698

[B58] Bournia VK Evangelou K Sfikakis PP Therapeutic inhibition of tyrosine kinases in systemic sclerosis: a review of published experience on the first 108 patients treated with imatinib Semin Arthritis Rheum 2013 42 377 390 10.1016/j.semarthrit.2012.06.001 22789835

[B59] Soldano S Montagna P Brizzolara R Ferrone C Parodio A Sulli A Seriolo B Villaggio B Cutolo M Endothelin receptor antagonists: effects on extracellular matrix synthesis in primary cultures of skin fibroblasts from systemic sclerosis patients Reumatismo 2012 64 326 334 23256109 10.4081/reumatismo.2012.326

